# Access of Syrian Refugee Women to Sexual and Reproductive Health Services in Turkey during COVID-19: Eray Ontas

**DOI:** 10.1093/eurpub/ckac129.254

**Published:** 2022-10-25

**Authors:** E Ontas, S Bahar Ozvaris, B Guciz Dogan, T Kutluk

**Affiliations:** 1 Zonguldak Provincial Health Directorate, Republic of Türkiye Ministry of Health, Zonguldak, Turkey; 2 Department of Public Health, Hacettepe University Faculty of Medicine, Ankara, Turkey; 3 Department of Pediatrics, Hacettepe University Faculty of Medicine, Ankara, Turkey

## Abstract

Women and girls are more disadvantaged in times of crisis, and their chances of surviving and fleeing are limited. As the Syrian crisis enters its eleventh year, Turkey alone hosts the largest population (over 3.7 million). It aimed to evaluate essential reproductive health services in the shadow of the pandemic that deepens the crisis. The research was conducted in April 2021 with 637 married Syrian refugee women aged 15-49 living in Ankara (mean age: 29.6). The median age at first marriage was 17, and consanguinity with her spouse was 29.8%. 8.6% were illiterate. 36.3% did not/could not benefit from public hospitals free of charge, and 89.8% did not have health insurance. 96.7% had a previous pregnancy (median:4). Since the pandemic's beginning, 35.6% have been pregnant (n = 219), and 14% are still pregnant (n = 86). Of 133 women whose pregnancies ended during this period, 78.2% gave birth (n = 104) and 21.8% miscarried. 12.8% of pregnancy was terminated at home (n = 17, 14 of which could not receive support from anyone). 41.1% of those who became pregnant during the pandemic were not followed up in pregnancy; 45.1% of those whose pregnancies ended were not followed up in the puerperium. 29.5% of all the participants stated that they had not used any birth control method. The most common reason for not using family planning is fear of harm to health and their spouse's disapproval. Only 3.3% of those currently using birth control methods stated difficulty accessing birth control methods. The most used information resources about pregnancy, childbirth and contraception methods were hospitals and Migrant Health Centres.

This study is funded through the UK Research and Innovation GCRF Research for Health In Conflict (R4HC-MENA), developing capability, partnerships and research in the Middle and Near East (MENA) [ES/P010962/1].

**Key messages:**

• In refugee crises, women's health should be structured as a separate heading in the health system and unmet sexual health needs should be met, especially in culture-oriented primary care services.

• Women should be empowered with a health system where women can determine their own needs and make decisions about their bodies.

